# Evaluating the immune responses of mice to subcutaneous immunization with *Helicobacter pylori* urease B subunit

**DOI:** 10.1186/2049-1891-5-14

**Published:** 2014-02-22

**Authors:** Peng Sun, Jia-Qi Wang, Yu-Tao Zhang, Sheng-Guo Zhao

**Affiliations:** 1 State Key Laboratory of Animal Nutrition, Institute of Animal Science, Chinese Academy of Agricultural Sciences, No.2 Yuanmingyuan West Road, Beijing 100193, P. R. China

**Keywords:** Antibody, Cytokines, *Helicobacter pylori* urease B subunit, Mice

## Abstract

**Background:**

*Helicobacter pylori*, a gram-negative bacterial pathogen that expresses a strong urease activity, is associated with the development of gastroduodenal disease. Urease B subunit, one of the two structural subunits of urease, was expressed in *E. coli* BL21 (DE3) strain. The objective of this study was to evaluate the effects of *Helicobacter pylori* urease B subunit on the immune responses in mice by subcutaneous immunization.

**Methods:**

The mice were immunized and boosted with *Helicobacter pylori* urease B subunit antigen subcutaneously three times with 2-wk intervals between the immunizations and boosters. The mice in the control group were immunized with PBS. The adjuvant group received PBS containing complete/incomplete freund’s adjuvant identical to antigen group without *Helicobacter pylori* urease B subunit antigen. Four weeks after the final booster, all the mice were sacrificed. Blood was collected on d 0, 14, 28 and 56 before immunization, booster and sacrifice, respectively. Immediately after sacrifice, gastric liquid and spleen were collected for antibody and cytokine analyses.

**Results:**

Urease B subunit increased the concentrations of serum and gastric anti-urease B antigen specific IgG, and the levels of interleukin-4 and interferon-γ in splenocytes of the mice (*P* < 0.05).

**Conclusions:**

This study demonstrated that recombinant urease B subunit can induce systemic and local immune responses in mice by subcutaneous immunization, which might be used as the effective component of vaccine against *Helicobacter pylori*.

## Introduction

*Helicobacter pylori*, a gram-negative bacterial pathogen that expresses a strong urease activity, is associated with the development of chronic gastritis, peptic ulceration and gastric carcinoma [1]. It is one of the most common bacteria that infect people and more than half of the worldwide population has been infected. Although the application of H2 antagonists, antibiotics and recently proton pump inhibitor (PPI) can temporarily eradicate *H. pylori* infection, negative effects concerning antibiotic-resistant strains always limit the treatment [2-4]. In case of the difficulty in completely eradicating *H. pylori* from the upper gastrointestinal tract of the patients, utilization of a safe and effective vaccine might be the ideal strategy to immunologically prevent *H. pylori* infection [5-7].

Urease, a recognized virulence factor of *H. pylori*, is expressed broadly on the surface of *H. pylori* and contributes a lot to colonization of *H. pylori*[8,9]. The main function of urease is to degrade urea resulting in the release of ammonia. It has been postulated that urease consists of two structural subunits, urease A and B. Previous studies concerning immunology indicate that urease B subunit is highly immunogenic which has been regarded as one of the most prospectively protective antigens [10].

Mucosal immunization with the attenuated *H. pylori* or other bacteria containing urease or its subunits antigens via oral, nasal, rectal or other routes has previously been reported as effective ways to protect human or animals from *H. pylori* infection [11-13]. However, the immunogenicity of the foreign antigens via mucosal routes is usually poor without the utilization of adjuvant [13]. In case of the safety and side effects of oral administration of antigens and adjuvant that might cause diarrhea and threaten the health of the host, parenteral immunization has been recommended by various recent studies which suggest that parenteral immunization can also protect body against *H. pylori* infection as effectively as oral immunization because of the stimulation of relatively higher specific antibody concentrations [1,11].

Here *H. pylori* urease B subunit was obtained after plasmid-encoded urease B from *H. pylori* was expressed in *E. coli* BL21 (DE3) strain. To evaluate the effects of *H. pylori* urease B subunit on the immune responses by subcutaneous immunization in mice, the indices of specific antibodies in serum and stomach as well as the splenocyte-secreted cytokines were determined in this study.

## Materials and methods

### Establishment and characterization of purified *H. pylori* urease B subunit

*E. coli* SE 5000 containing the urease expression genes used in this study were kindly provided by H. T. Mobley from Department of Biology, Kilgore College, Kilgore, TX, USA. *E. coli* BL21 (DE3) strain was used as a recipient for the recombinant urease plasmid constructs that expressed urease B from the plasmid in which urease B genes from *H. pylori* had been cloned. After the *E. coli* BL21 (DE3) strain containing the urease B genes was activated overnight at 37°C, the bacteria were inoculated and cultured in Luria broth medium. Then the culture was induced by isopropyl-β-D-thiogalactopyranoside (IPTG; Sigma) and centrifuged at 6,000 × *g* for 15 min. Cells were harvested and catabolizated by lysozyme and nuclease. After sonication, the cell lysate was centrifuged at 12,000 × *g* for 10 min. Urease B was expressed and obtained after washing with Buffer B (5% triton, 50 mol/L Tris–HCl, 50 mmol/L NaCl, and 5 mmol/L EDTA) and resolving in urea-Tris–HCl solution. Urease B antigen was purified by using a Ni-NTA kit (Novagen, Madison, WI) according to manufacturer’s instructions. Sodium dodecyl sulfate polyacrylamide gel electrophoresis (SDS-PAGE) was applied to identify the expressed protein and the purity of urease B was measured by Bradford method (Bio-Rad, Hercules, CA).

### Mice

All the mice used in this experiment were maintained in accordance with the principles of Chinese Academy of Agricultural Sciences Animal Care and Use Committee. Fifteen specific-pathogen-free, six-week-old female Kunming mice weighing 17–20 g were purchased from the Beijing Laboratory Animal Research Centre (Beijing, China) and divided into three treatments at random. Each treatment had five replicates with one mouse per replicate. All animals were housed in plastic cages in a mechanically ventilated nursery room where 12 h light: 12 h dark was set, and constant temperature remained at 23–25°C and relative humidity at 50-60%. All the mice had sterilized commercial chow (Beijing Laboratory Animal Research Centre) and water *ad libitum* under pathogen-free conditions.

### Immunization procedures and sample collection

Three groups of 5 mice were used as control, adjuvant and antigen. After seven days of adaptation, mice were immunized and boosted subcutaneously three times with 2-wk intervals between the immunizations and booster. *H. pylori* urease B subunit antigen was mixed with complete/incomplete freund’s adjuvant (v/v, 1:1), and 40 μg of *H. pylori* urease B subunit antigen in a volume of 100 μL of emulsion was injected into the lower back of the antigenic mice on d 0 and 14. The mice in the control group were immunized with PBS. The adjuvant group received PBS containing complete/incomplete freund’s adjuvant identical to antigen group without *H. pylori* urease B subunit antigen. The mice were boosted with 80 μg of urease B antigen and incomplete freund’s adjuvant on d 28. Blood was collected retro-orbitally before immunization and booster on d 0, 14 and 28, respectively.

Four wk after the final booster, all the mice were sacrificed after final blood collection from the heart. Mice were immersed into 75% ethanol for 5 min immediately after sacrifice. Thereafter, the peritoneal cavity was opened, and the spleen was removed from each mouse followed by the recovery of gastric fluid flushed with 1 mL of phosphate buffered saline (PBS) containing protease inhibitor.

### Spleen cell culture

Immediately after sacrifice, the spleen was aseptically removed from mice and the tissue was minced by syringe and washed twice with RPMI 1640 containing 10% fecal bovine serum (HyClone Laboratories Inc. Logan UT), 10 mmol/L Hepes, 100 μg/mL penicillin and 100 μg/mL streptomycin (Sigma, St. Louis, MO). After erythrocyte lysis, splenocytes were separated and cultured in 24-well plates at a density of 1 × 10^6^ cells per well with or without 10 μg/mL *H. pylori* lysate for 48 h at 37°C and 5% CO_2_. The supernatant was harvested on d 0, 7 and 14 and stored at −70°C for cytokine assay.

### Measurement of serum and gastric antibodies by ELISA

The gastric fluid sample was centrifuged at 10,000 × *g* at 4°C for 10 min, and the supernatant was obtained for IgA and IgG determination. The measurement of serum anti- *H. pylori* urease B specific IgA, IgG, IgE, gastric IgA and IgG levels was performed by enzyme-linked immunosorbent assay (ELISA) using an indirect ELISA as described by Weltzin et al. [13] with some modifications. A 96 well microtiter plate (Costar, Corning Incorporated, Corning, NY, USA) was coated overnight at 4°C with 4.0 μg/mL urease B in carbonate buffer (NaHCO_3_/Na_2_CO_3_, pH 9.6) and then free binding sites were blocked with PBS containing 1% bovine serum albumin (BSA) and 0.1% Tween 20 (ELISA buffer) for 1 h at 37°C. Duplicate serum and gastric fluid samples were diluted in ELISA buffer and incubated for 2 h at 37°C. The plates were then incubated with ELISA buffer containing IgA, IgG or IgE antibodies conjugated with horseradish peroxidase (HRP; Serotec, Oxford, England) for 1 h at 37°C. Samples were washed five times with PBS containing 0.1% Tween 20 between each incubation step. Then the plates were developed with tetramethylbenzidine (TMB) to measure the absorbance at 450 nm. The data were expressed as optical density (OD) units.

### Determination of cytokines in the cultured splenocyte supernatant

Single splenic cell suspension was inoculated *in vitro* with or without *H. pylori* lysate at the concentration of 10 μg/mL in a total volume of 1 mL. The supernatants were collected on d 0, 7 and 14, respectively. Interleukin-4 (IL-4) and interferon-γ (IFN-γ) concentrations in the cultured splenocyte supernatant were measured by the mouse ELISA Kit (Jingmei Biotech, Shanghai, China) following the manufacturer’s instructions.

### Statistical analysis

All data were analyzed using the ANOVA procedure of SAS system (version 8.2, SAS Institute, Inc., Cary, NC, USA). *P* values less than 0.05 were considered statistically significant.

## Results

### Expression of urease B subunit in *E. coli*

**Figure 1:**
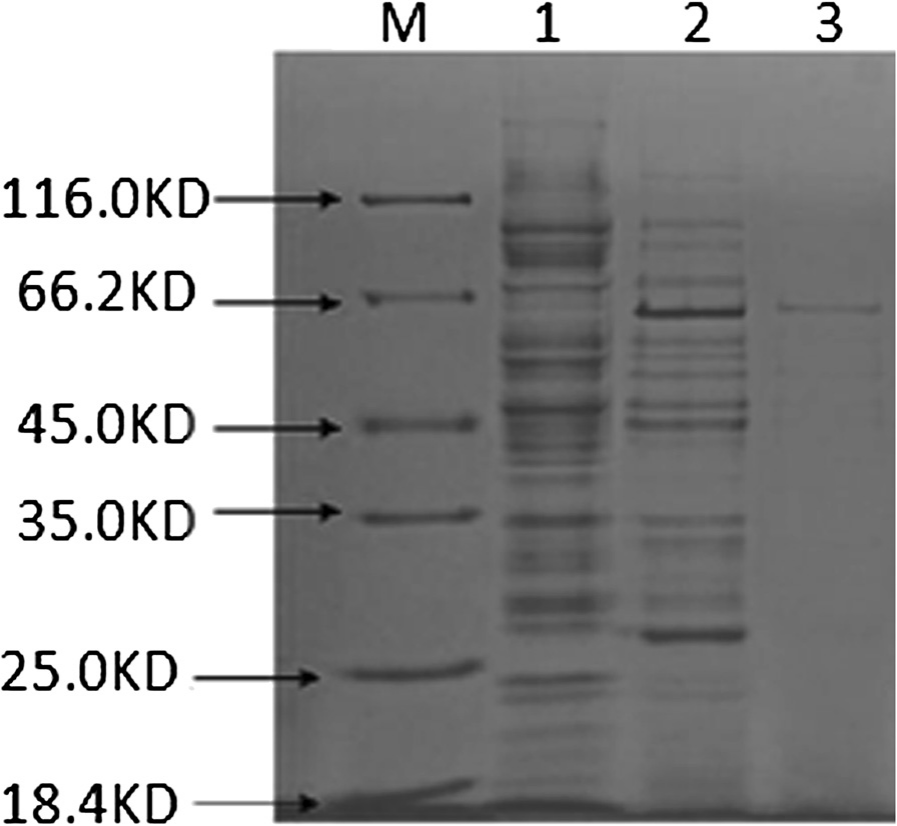
**SDS-PAGE analysis of the recombinant urease B subunit.** The protein was analyzed on a 12% SDS-PAGE. Lane M, low molecular weight standard; lanes 1 and 2, lysates of E. coli cells harboring urease B subunit before and after IPTG induction; lane 3, purified recombinant urease B subunit protein.

*E. coli* BL21 (DE3) strain was assayed using SDS-polyacrylamide gels for expression of urease B. As shown in Figure 1, protein of 64 KD was observed followed by Coomassie Brilliant Blue (CBB) R-250 staining, which was well correlated with urease B. The purity of urease B was measured and the concentration was 0.40 mg/mL. The results of our present study suggest that urease B subunit from *H. pylori* can be obtained through mostly expression in *E. coli* without negative effects and is at least suitable for animal protection experiments in further studies.

### Strong antibody responses to recombinant urease B subunit after its subcutaneous delivery with complete/incomplete freund’s adjuvant

**Figure 2:**
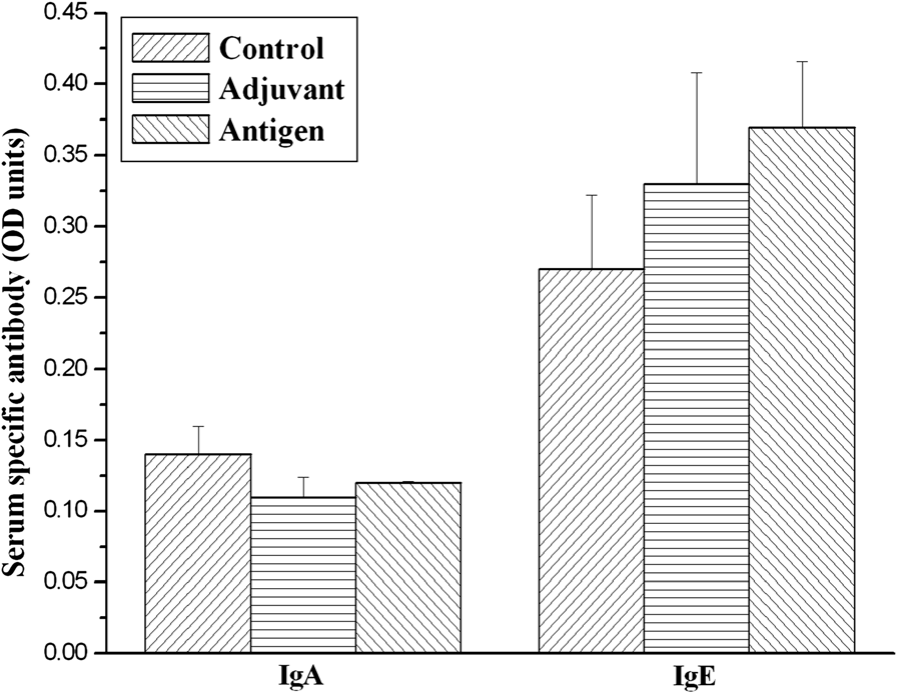
**Urease B subunit specific IgA and IgE in sera of mice immunized and boosted subcutaneously with PBS (Control), complete/incomplete freund’s adjuvant (Adjuvant) and urease B subunit antigen containing complete/incomplete freund’s adjuvant (Antigen).** Sera were collected on d 56 and tested for urease B subunit specific antibody by ELISA. Each data point is presented as the groups mean ± SEM of 5 replicates per treatment.

Mice were immunized and boosted with recombinant urease B subunit and freund’s adjuvant by subcutaneous route. To investigate whether urease B subunit expressed by *E. coli* was capable of inducing specific antibody responses and what type of antibody production against urease B was stimulated by systemic immunization, specific IgA, IgE and IgG in the serum and IgA and IgG in gastric liquid were measured by ELISA. It is reported that parenteral immunization tends to induce low levels of secretary IgA but often increase a low level IgE production in most mice [13]. As presented in Figure 2, low levels of serum IgA to urease B were generated by subcutaneous immunization with recombinant urease B subunit, which is consistent with published data [1]. Serum IgE was stimulated slightly with the subcutaneous immunization (Figure 2), but no difference was observed among the three groups.

**Figure 3:**
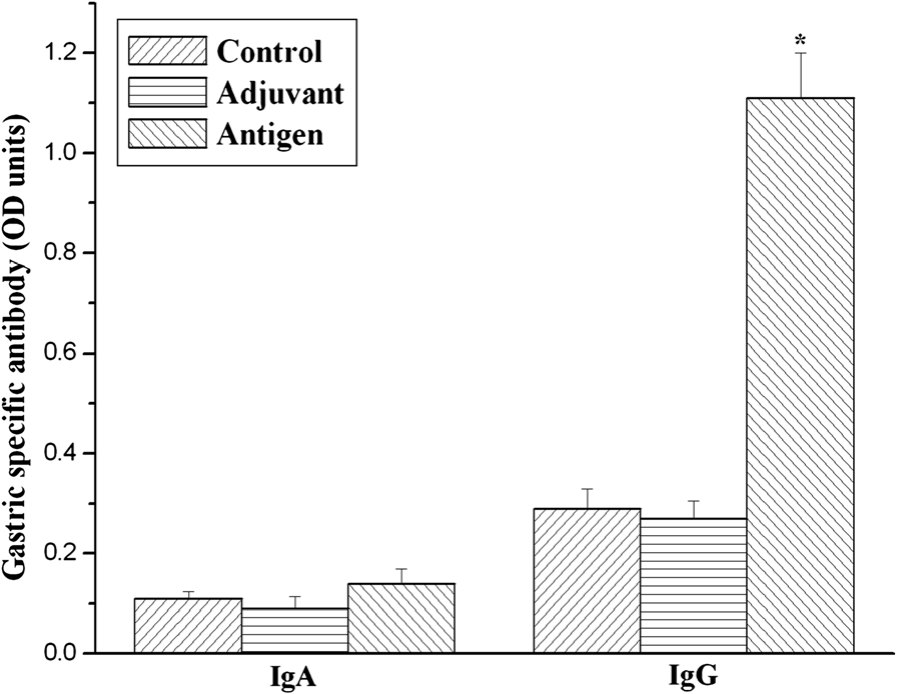
**Urease B subunit specific IgG in sera of mice immunized and boosted subcutaneously with PBS (Control), complete/incomplete freund’s adjuvant (Adjuvant) and urease B subunit antigen containing complete/incomplete freund’s adjuvant (Antigen).** Sera were collected on day 0, 14, 28 and 56 and tested for urease B subunit specific antibody by ELISA. Each data point is presented as the groups mean ± SEM of 5 replicates per treatment. *P < 0.05 vs. control.

**Figure 4:**
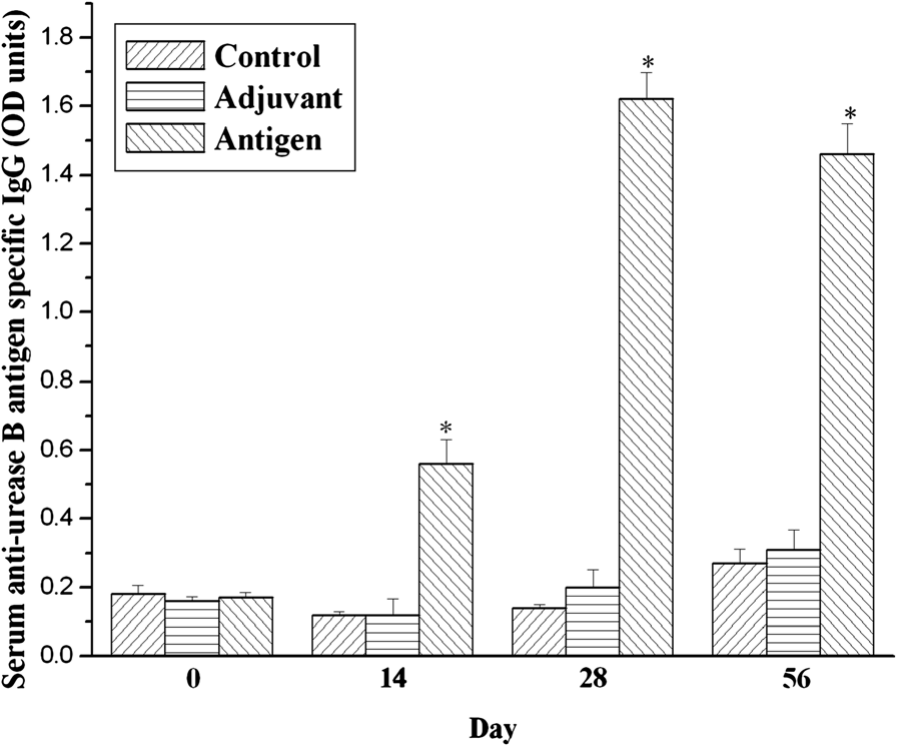
**Urease B subunit specific IgA and IgG in the gastric liquid of mice immunized and boosted subcutaneously with PBS (Control), complete/incomplete freund’s adjuvant (Adjuvant) and urease B subunit antigen containing complete/incomplete freund’s adjuvant (Antigen).** Gastric fluid samples were collected on day 56 and tested for urease B subunit specific antibody by ELISA. Each data point is presented as the groups mean ± SEM of 5 replicates per treatment. *P < 0.05 vs. control.

As shown in Figure 3, IgG specific for urease B was nearly zero in the serum of unimmunized mice. Nevertheless, serum urease B antigen-specific IgG were significantly increased on day 14, 28 and 56 in the antigen immunized group compared with the control and adjuvant injected groups (*P* < 0.05). Furthermore, gastric IgG to urease B subunit antigen was statistically higher (*P* < 0.05) in urease B antigen-immunized mice than in the control and adjuvant-injected mice (Figure 4), although gastric anti-urease B antigen specific IgA was not influenced among the three groups.

### Subcutaneous immunization with recombinant urease B subunit results in elevated Th1/Th2 responses

**Figure 5:**
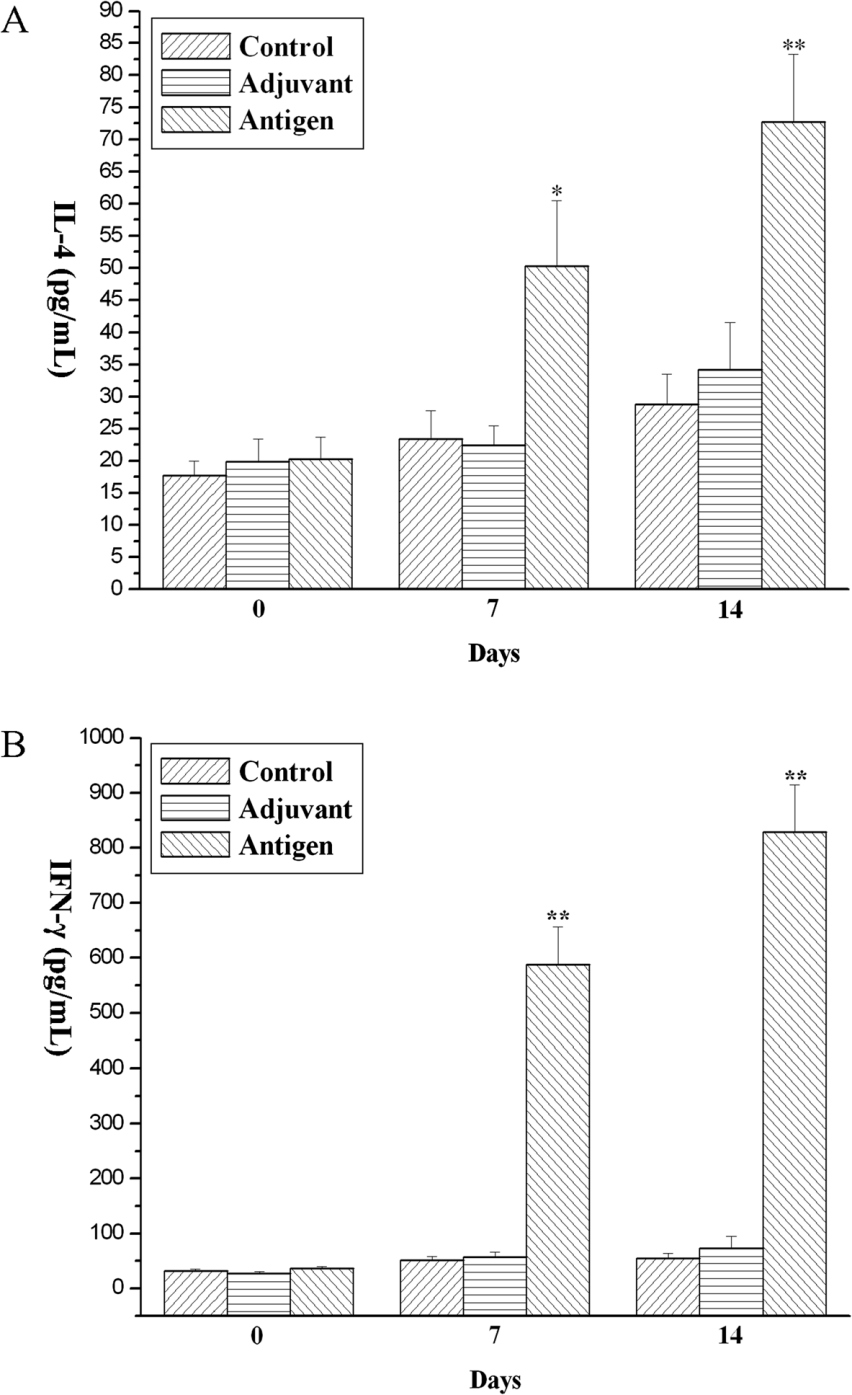
**The IL-4 (A) and IFN-γ (B) production in *H. pylori *lysate-stimulated splenocytes at day 0, 7, and 14 after sacrifice from mice immunized and boosted subcutaneously with PBS (Control), complete/incomplete freund’s adjuvant (Adjuvant) and urease B subunit antigen containing complete/incomplete freund’s adjuvant (Antigen).** Each data point is presented as the groups mean ± SEM of 5 replicates per group. *P < 0.05 vs. control, **P < 0.01 vs. control.

Available evidence has revealed that systemic immunization can protect mice against *H. pylori* infection [1,13,14]. But it is still controversial whether Th1 cells, Th2 cells or both the two types of cells contribute to the protection. To further investigate the function of Th1/Th2 cells under the conditions of our present study, we determined IL-4 and IFN-γ that has been regarded as the two representative interleukins of Th1 and Th2 type immune responses [15,16] to indirectly reflect the function of Th1 and Th2 immune responses in the protection against *H. pylori* infection. As shown in Figure 5A, concentrations of IL-4 increased progressively in the supernatant of spleen cell culture of urease B immunized mice compared to the PBS control and adjuvant control groups (*P* < 0.05). Simultaneously, the production of IFN-γ was also enhanced by splenic cells while the concentrations of IFN-γ secteted by splenocytes from control mice or adjuvant mice were very low and nearly zero (Figure 5B).

## Discussion

The present study evaluated the effects of *H. pylori* urease B subunit on the immune responses of mice by subcutaneous immunization. Colonization in the stomach mucosa by *H. pylori* causes the incidence of gastrointestinal diseases for adults and children. Although it is easy to prevent the growth of *H. pylori* by antibiotics *in vitro*, eradication of this bacterium from the infectious stomach of patients is of great difficulty for the existence of antibiotic resistance during therapy as well as re-infection due to the prevalence of infection [17,18]. Most investigators have focused on the prophylactic and/or curative immunization using *H. pylori* sonicates or recombinant urease, especially urease B as oral vaccines for the protection against *H. pylori* infection [1,5,6]. Considering the infectious objectives by *H. pylori* are the mucosa of gastrointestine, immunization through mucosal routes, such as oral immunization has been widely adopted to trigger and stimulate the immune system underlying the intestinal mucosa in a variety of previous researches which tended to enhance IgA production [2,5,8]. Although IgA has been found to play an important role in mucosal immunity against invasion by mucosal pathogens, some investigations have demonstrated that secretory IgA is not required for protection of *H. pylori* infection because immunoglobulin-deficient mice can also be protected when lack of antibody responses [6,11,19,20]. Consistent with those reports, little or no specific IgA to urease B subunit in gastric liquid was detected in the three groups in the present study.

As mentioned above, one of the major drawbacks of oral immunization is the lower immunogenicity of the ingested foreign antigens which cannot stimulate stronger immune responses in the body unless some adjuvants are used [13]. Compared to oral immunization, parenteral immunization avoids the toxicity and diarrhea caused by the contact of foreign antigens and adjuvants to the intestinal epithelial cells [13]. Furthermore, parenteral delivery of antigens can induce much stronger humoral and systemic immune responses. To date, many studies have revealed that parenteral immunization is capable of protecting against *H. pylori* booster better than or as effectively as mucosal immunization [1,11,21-23]. Therefore, subcutaneous immunization was adopted in the present study to evaluate the effects of *H. pylori* urease B subunit in a mouse model.

Although previous publications have shown that antibodies are helpful but not essential for host immune protection [19], antibodies can clear most infections and are beneficial for the host [7]. Even if antibodies play limited role in protection from *Helicobacter* infection, they are still the markers of immune responses and other arms of immune system may function importantly [7]. It is well known that IgG is the main antibody in the serum stimulated by foreign antigens especially via parenteral immunization [24,25]. In the current study, anti-urease IgG antibodies in the serum and gastric liquid were significantly increased indicating that urease B subunit delivered by *E. coli* is highly immunogenic and capable of inducing protection against *H. pylori*. It has been well postulated that IgG is sufficient to confer protection against a variety of infections, including mucosal infectious diseases [26]. The IgG antibody plays an essential role in the local and systemic immune responses during the protection from *Helicobacter* infection, which has been reported in numerous publications [6,26,27].

It has been well documented that T lymphocytes can be classified as CD4^+^ or CD8^+^ subsets differentiated with their surface markers [28]. Recently, another T lymphocyte subset, that is, Th17 cells has been discovered [29,30]. CD4^+^ T cells are defined as helper T cells. T-helper 1 (Th1) cells, Th2 cells and Th17 cells are different types of helper T cells resulting in the secretion of different patterns of cytokines [15,29,31]. Th1 lymphocytes are characterized by the secretion of IL-2, IFN-γ and so on, which are vital to cell-mediated immunity, while Th2 lymphocytes are predominantly involved in humoral immunity and allergic responses leading to the production of IL-4, IL-5 and IL-10, etc. [15,16]. Th 17 cells produce IL-17, IL-6, TNF-α and IL-22, etc. [29].

Previous studies have proven that IL-4 is a Th2-type representative cytokine which plays a key role in allergic inflammation [16]. Production of high level of IL-4 may indicate preferential activation of Th2 cells. However, IFN-γ is the essential Th1 cytokine that functions reciprocally on the induction of IL-4 [32]. The secretion of Th1/Th2 cytokines may indirectly reflect the balance of Th1/Th2 type immune responses in the body. Gottwein et al. [10] presented that immunization with *Helicobacter* antigens and Freund’s adjuvant induced protective anti-*Helicobacter* immunity resulting in production of IFN-γ. The analysis for cytokines in the cultured splenocyte supernatant also showed that concentrations of IL-4 and IFN-γ was significantly elevated in the supernatant of spleen cell culture of urease B immunized mice compared to the PBS control and adjuvant control groups, which suggested that Th1/Th2 type responses were both stimulated by the recombinant urease B subunit antigen. Similar results were also obtained by Guy et al. [1] who found that a combination of strong Th1 and Th2 responses induced by urease mixed with adjuvants elicited better protection from *H. pylori* infection than that of a predominantly Th2 type response.

## Conclusions

The current study demonstrates that recombinant urease B subunit induced higher concentrations of serum and gastric IgG as well as an increase of IL-4 and IFN-γ in splenocytes of the immunized mice by subcutaneous immunization. The application of urease B subunit in parenteral inoculation strategies might enlighten us to use it as the effective component of vaccine against *H. pylori*.

## Competing interests

The authors declare that they have no competing interests in relation to this study.

## Authors’ contributions

PS carried out the experiment and drafted the manuscript. JQW conceived the study, participated in its design and coordination, and helped draft the manuscript. YTZ performed the animal experiments. All authors read and approved the final manuscript.

## References

[B1] Guy B Hessler C Fourage S Haensler J Vialon-Lafay E Rokbi B Millet MQ Systemic immunization with urease protects mice against *Helicobacter pylori* infection Vaccine 1998 16 850 856 10.1016/S0264-410X(97)00258-2 9627943

[B2] Marchetti M Aricò B Burroni D Figura N Rappuoli R Ghiara P Development of a mouse model of *Helicobacter pylori* infection that mimics human disease Science 1995 267 1655 1658 10.1126/science.7886456 7886456

[B3] Thibault A Marteau P Drouet L Slove AL Sollier CBD Camus M Dray X In vivo effect of proton-pump inhibitors on gastric plasmin-dependent fibrinolysis: a study in a porcine model Digest Liver Dis 2012 44 995 998 10.1016/j.dld.2012.06.017 22890053

[B4] Kumar KR Iqbal R Coss E Park C Cryer B Genta RM Helicobacter gastritis induces changes in the oxyntic mucosa indistinguishable from the effects of proton pump inhibitors Hum Pathol 2013 44 2706 2710 10.1016/j.humpath.2013.07.015 24071014

[B5] Czinn SJ Nedrud JG Oral immunization against *Helicobacter pylori* Infect Immun 1991 59 2359 2363 2050403 10.1128/iai.59.7.2359-2363.1991 PMC258018

[B6] Dubois A Lee CK Fiala N Kleanthous H Mehlman PT Monath T Immunization against natural *Helicobacter pylori* infection in nonhuman primates Infect Immun 1998 66 4340 4346 9712786 10.1128/iai.66.9.4340-4346.1998 PMC108524

[B7] Bégué RE Sadowska-Krowicka H Protective efficacy of recombinant urease B and aluminum hydroxide against *Helicobacter pylori* infection in a mouse model FEMS Immunol Med Microbiol 2010 60 142 146 10.1111/j.1574-695X.2010.00726.x 20731722 PMC2970735

[B8] Gómez-Duarte OG Lucas B Yan ZX Panthel K Haas R Meyer TF Protection of mice against gastric colonization by *Helicobacter pylori* by single oral dose immunization with attenuated *Salmonella typhimurium* producing urease subunits A and B Vaccine 1998 16 460 471 10.1016/S0264-410X(97)00247-8 9491500

[B9] Wu C Zou QM Guo H Yuan XP Zhang WJ Lu DS Mao XH Expression, purification and immune-characteristics of recombination UreB protein of *H. pylori* World J Gastroentero 2001 7 389 393 10.3748/wjg.v7.i3.389 PMC4688728 11819796

[B10] Pappo J Thomas WD Kabok Z Taylor NS Murphy JC Fox JG Effect of oral immunization with recombinant urease on murine *Helicobacter felis* gastritis Infect Immun 1995 63 1246 1252 7890380 10.1128/iai.63.4.1246-1252.1995 PMC173142

[B11] Ermak TH Giannasca PJ Nichols R Myers GA Nedrud J Weltzin R Lee CK Kleanthous H Monath TP Immunization of mice with urease vaccine affords protection against *Helicobacter pylori* infection in the absence of antibodies and is mediated by MHC-class II-restricted responses J Exp Med 1998 188 2277 2288 10.1084/jem.188.12.2277 9858514 PMC2212427

[B12] Weltzin R Kleanthous H Guirakhoo F Monath TP Lee CK Novel intranasal immunization techniques for antibody induction and protection of mice against gastric *Helicobacter felis* infection Vaccine 1997 15 370 376 10.1016/S0264-410X(97)00203-X 9141207

[B13] Weltzin R Guy B Thomas WD Giannasca PJ Monath TP Parenteral adjuvant activities of *Escherichia coli* heat-labile toxin and its B subunit for immunization of mice against gastric *helicobacter pylori* infection Infect Immun 2000 68 2775 2782 10.1128/IAI.68.5.2775-2782.2000 10768972 PMC97487

[B14] Gottwein JM Blanchard TG Targoni OS Eisenberg JC Zagorski BM Redline RW Nedrud JG Tary-Lehmann M Lehmann PV Czinn SJ Protective anti-*Helicobacter* immunity is induced with aluminum hydroxide or complete Freund’s adjuvant by systemic immunization J Infect Dis 2001 184 308 314 10.1086/322032 11443556

[B15] Carter LL Dutton RW Type 1 and Type 2: a fundamental dichotomy for all T-cell subsets Curr Opin Immumol 1996 8 336 342 10.1016/S0952-7915(96)80122-1 8793995

[B16] Prescott VE Hogan SP Genetically modified plants and food hypersensitivity diseases: Usage and implication of experimental models for risk assessment Pharmacol Therapeut 2006 111 374 383 10.1016/j.pharmthera.2005.10.005 16364445

[B17] Schutze K Hentschel E Dragosics B Hirscl AM *Helicobacter pylori* reinfection with identical organisms: transmission by the patients’ spouses Gut 1995 36 831 833 10.1136/gut.36.6.831 7615268 PMC1382617

[B18] Xia HX Windle HJ Marshall DG Smith CJ Keane CT Recrudescence of *Helicobacter pylori* after apparently successful eradication: novel application of randomly amplified polymorphic DNA fingerprinting Gut 1995 37 30 34 10.1136/gut.37.1.30 7672675 PMC1382763

[B19] Blanchard TG Czinn SJ Redline RW Sigmund N Harriman G Nedrud JG Antibody-independent protective mucosal immunity to gastric helicobacter infection in mice Cell Immunol 1999 191 74 80 10.1006/cimm.1998.1421 9918689

[B20] Pappo J Torrey D Castriotta L Savinainen A Kabok Z Ibraghimov A *Helicobacter pylori* infection in immunized mice lacking major histocompatibility complex class I and class II functions Infect Immun 1999 67 337 341 9864234 10.1128/iai.67.1.337-341.1999 PMC96315

[B21] Eaton KA Krakowka S Chronic active gastritis due to *Helicobacter pylori* in immuized gnotobiotic piglets Gastrienterology 1992 103 1580 1586 10.1016/0016-5085(92)91181-3 1426878

[B22] Eaton KA Ringler SS Krakowka S Vaccination of gnotobiotic piglets against *Helicobacter pylori* J Infect Dis 1998 178 1399 1405 10.1086/314463 9780261

[B23] Guy B Hessler C Fourage S Rokbi B Millet MJQ Comparison between targeted and untargeted systemic immunizations with adjuvanted urease to cure *Helicobacter pylori* infection in mice Vaccine 1999 17 1130 1135 10.1016/S0264-410X(98)00332-6 10195624

[B24] Helm RM Furuta GT Stanley JS Ye JH Cockrell G Connaughton C Simpson P A neonatal swine model for peanut allergy J Allergy Clin Immun 2002 109 136 142 10.1067/mai.2002.120551 11799380

[B25] You JM Li DF Qiao SY Wang ZR He PL Ou DY Dong B Development of monoclonal antibody-based competitive ELISA for detection of β-conglycinin, an allergen from soybean Food Chem 2008 106 352 360 10.1016/j.foodchem.2007.05.040

[B26] Robbins JB Schneerson R Szu SC Perspective: Hypothesis: Serum IgG antibody is sufficient to confer protection against infectious diseases by inactivating the inoculum J Infect Dis 1995 171 1387 1398 10.1093/infdis/171.6.1387 7769272

[B27] Ferrero RL Thiberge J Labigne A Local immunoglobulin G antibodies in the stomach may contribute to immunity against *Helicobacter* infection in mice Gasteroenterology 1997 113 185 194 10.1016/S0016-5085(97)70094-5 9207277

[B28] Swain S T cell subsets and the recognition of MHC class Immunol Rev 1983 74 129 142 10.1111/j.1600-065X.1983.tb01087.x 6226585

[B29] Ciprandi G Filaci G Battaglia F Fenoglio D Peripheral Th-17 cells in allergic rhinitis: New evidence Int Immunopharmacol 2010 10 226 229 10.1016/j.intimp.2009.11.004 19925886

[B30] Bettelli E Oukka M Kuchroo VK T(H)-17 cells in the circle of immunity and autoimmunity Nat Immunol 2007 8 345 350 17375096 10.1038/ni0407-345

[B31] Chen DY Hua Q Mao CM Jiao ZJ Wang SJ Yu LC Ying X Dai DF Yin LJ Xu HX Increased IL-17-producing CD4+ T cells in patients with esophageal cancer Cell Immunol 2012 272 166 174 10.1016/j.cellimm.2011.10.015 22082565

[B32] Snapper CM Paul WE Interferon-γ and B cell stimulatory factor-1 reciprocally regulate Ig isotype production Science 1987 236 944 947 10.1126/science.3107127 3107127

